# The role of local health officers in advancing public health and primary care integration: lessons from the ongoing Universal Health Coverage reforms in the Philippines

**DOI:** 10.1136/bmjgh-2023-014118

**Published:** 2024-01-23

**Authors:** Vergil de Claro, Juan Bernardo Lava, Clemencia Bondoc, Laurentiu Stan

**Affiliations:** 1 RTI International, Pasig City, Philippines; 2 Zarraga Municipal Health Office, Association of Municipal Health Officers, Zarraga, Iloilo, Philippines

**Keywords:** Public Health, Health systems

## Abstract

The COVID-19 pandemic has highlighted the persistent fragmentation of health systems and has amplified the necessity for integration. This issue is particularly pronounced in decentralise settings, where fragmentation is evident with poor coordination that impedes timely information sharing, efficient resource allocation and effective response to health threats. It is within this context that the Philippine Universal Health Care law introduced reforms focusing on equitable access and resilient health systems through intermunicipal cooperation, enhancing primary care networks and harnessing digital health technologies—efforts that underline the demand for a comprehensively integrated healthcare system. The WHO and the global community have long called for integration as a strategy to optimise healthcare delivery. The authors contend that at the core of health system integration lies the need to synchronise public health and primary care interventions to enhance individual and population health. Drawing lessons from the implementation of a pilot project in the Philippines which demonstrates an integrated approach to delivering COVID-19 vaccination, family planning and primary care services, this paper examines the crucial role of local health officers in the process, offering insights and practical lessons for engaging these key actors to advance health system integration. These lessons may hold relevance for other low-ncome and middle-income economies pursuing similar reforms, providing a path forward towards achieving universal health coverage.

**SUMMARY BOX**

- The persistent challenges brought about by health system fragmentation and the recent COVID-19 experience highlighted the urgency of examining how public and primary care interventions can be delivered more effectively and in an integrated manner.
- We describe an integrated approach to health service provision that was codesigned, managed and implemented by local health officers within a decentralized setting.
- Our paper demonstrates how the designated role of local health officers strategically positions them as the crucial link to integration, effectively bridging the current gaps in implementation.
- We outline how the role and expertise of local health officers can be harnessed to support integrated care. Our recommendations encompass enhancing their capabilities, designing incentives, defining their roles within a collaborative framework, and incorporating technology-driven monitoring to facilitate decision-making.

## Introduction

The push factors for integration in health systems have become increasingly evident in light of experiences of fragmentation and the challenges exposed by the COVID-19 pandemic.^1 2^ In the context of decentralisation, fragmented systems emerge when different regions or local entities possess varying capacities and resources to deliver healthcare services leading to disparities in access, quality and outcomes. As a result, the COVID-19 pandemic has unravelled the vulnerabilities of a fragmented health system and underscored the importance of robust primary healthcare (PHC). As public health researchers and practitioners supporting health systems strengthening work in the Philippines, we have witnessed how a devolved setup already hampered by fragmentation^3^ and inadequate coordination and communication among healthcare entities, impedes the timely sharing of information, hinders resource allocation and weakens the overall response capacity to health threats.^4^ These factors have contributed to difficulties in identifying, monitoring and managing outbreaks at the community level, which are crucial for early detection and containment of infectious diseases. Addressing the inefficiencies of fragmented systems and bolstering primary care are vital steps towards achieving universal health coverage (UHC) and ensuring equitable access to quality healthcare services.^5^

Current efforts in many countries demonstrate the commitment to forge a more integrated and coordinated system of care, paving the way for a stronger and more resilient healthcare system for the benefit of all.^5^ For instance, the Philippine Universal Health Care law aims to ensure equitable access to quality healthcare for all its citizens and has embarked on implementing a comprehensive healthcare reform to address the existing gaps and build resilient health systems capable of withstanding future challenges. The reforms encompass strategies for promoting intermunicipal cooperation, strengthening primary care networks, enhancing health workforce capacity and leveraging digital health technologies to facilitate information exchange and coordination^6^—efforts that underline the demand for a healthcare system that is effectively integrated.

The WHO and the global community have long called for integration as a means to optimise healthcare delivery.^7^ But what is being integrated and how? The concept has been defined in a number of ways^8–10^ but a common feature is the ‘coming together of healthcare organisations into team-like configurations’ such as network-type structures, nationally unifying health systems (ie, reforms in funding and service delivery mechanisms) and boundary-spanning relationships (ie, public–private partnerships).^11^ The authors adopted the definition of integration as ‘a coherent set of methods and models on the funding, administrative, organisational, service delivery and clinical levels designed to create connectivity, alignment and collaboration within and between the cure and care sectors’.^10^ This definition denotes the occurrence of integration at various levels both at the organisational and programme or service levels^12^ which resonates with the ongoing efforts in the Philippines.

We believe that at the core of health system integration lies the need for public health and primary care interventions to work together, which is deemed as the next logical progression in the healthcare system. In its recent guidance document, the WHO emphasised the necessity of a robust and proactive public health function within primary care. This step is seen as crucial in safeguarding both individual and population health, promoting well-being and preventing diseases.^13^ An integrated delivery system represents an important step in the right direction to address the longstanding fragmentation in healthcare delivery.^14^

Seizing the opportunity presented by an enabling policy environment and intensified government response to COVID-19, we piloted an integrated approach to deliver COVID-19 vaccination, family planning and primary care services in Iloilo Province, Philippines from October 2022 to April 2023.^15^ Our objective was to derive valuable insights from the implementation research that can guide policy-makers and implementers to effectively integrate public health and primary care services. The project was carried out in collaboration with the provincial government, local health officers (provincial, city and municipal health officers), national and local officials from the Department of Health, and the public health insurance system, involving their active participation in its design, implementation and evaluation.

In this article, we focus on the crucial role played by local health officers in integrating public health and primary care. We reviewed the project implementation reports and the documentation from pause-and-reflect sessions to gain insights into their effective involvement and briefly explored literature to complement our findings. Drawing from the project’s experience and the literature review, we offer practical lessons on successfully engaging local health officers. These lessons can serve as valuable guidance for low-income and middle-income countries (LMICs) economies in decentralised settings pursuing similar reforms to advance health system integration as a key strategy for achieving UHC.

## From decentralisation to integration

Healthcare service delivery in the Philippines has been devolved to the local government units (LGUs) with the enactment of the Local Government Code in 1991. The legislation transferred specific health functions and responsibilities from the central government to provinces, cities and municipalities each conferred with power and authority to carry them out (box 1). The policy was considered to be one of the most radical and far-reaching decentralisation work with broad implication for other LMICs.^16^ While the health devolution in the Philippines yielded some benefits such as a more nuanced understanding of local health preferences thereby facilitating better alignment of resources with community needs, and fostering increased accountability and local participation,^16 17^ it encountered significant drawbacks at the health service delivery level. Contrary to expectations, the decentralisation did not lead to a more efficient and equitable distribution of resources nor did it result in greater health improvements compared with what could have been achieved under a centralised structure.^16 18^

Box 1 **Devolved health functions by type of local government unit^37^**

**Province**

- Health services which include hospitals and other tertiary health services.

**Municipality**

- Health services which include the implementation of programmes and projects on primary care, maternal and child care, and communicable and non-communicable disease control services.
- Access to secondary and tertiary health services.
- Purchase of medicines, medical supplies and equipment needed to carry out the services enumerated.

**City**

- All the services and facilities of the municipality and province.

**Barangay**

- Health and social welfare services which include maintenance of barangay health centre and daycare centre.
- Services and facilities related to general hygiene and sanitation, beautification and solid waste collection.

The inherent nature of health, transcending geographical boundaries, necessitates a coordinated effort among local governments—an aspect challenging to achieve in a decentralised setting. With decision-making vested in local authorities, priorities diverge, with health often relegated amid competition with other local development concerns. Additionally, certain health programmes, viewed as politically sensitive (eg, family planning) face resistance from influential community voices like the Catholic Church. This persistent fragmentation has led to consequences including weakened supervision of the local system by regional and national bodies, a fractured health information system impacting local and national policy-making, limitations on career paths for health workers confined within political boundaries, and altered the incentive structure for local governments, hindering the provision and financing of health services.^19^

Under this devolved set-up, the local health officers were tasked to perform multiple responsibilities, including governance and leadership, planning and monitoring, service delivery, disease surveillance, advocacy, and community empowerment. Some of these functions can be situated either in the public health or primary care domains while others belong to both, showing some level of interconnection and indicating areas that hold the greatest potential for integration (box 2).

Box 2 **Functions of local health officers under the Philippine Local Government Code^38^**

As prescribed under Republic Act No. 7160, the role of local health officers encompasses a wide spectrum of responsibilities, all directed towards fostering public health and well-being within their local jurisdiction.

1. Administrative leadership: Oversees the health services office, managing its staff, and creating guidelines, programme implementation protocols, and operational regulations to support the local chief executive’s efficient and effective execution of a health services programme directed towards implementing health-related projects and activities.
2. Advisory and technical support: Develops proposals for the local legislative council’s consideration and extend technical guidance and aid to the governor or mayor in facilitating actions to guarantee the provision of essential services and the establishment of sufficient facilities concerning health services.
3. Planning and implementation: Develop comprehensive plans and strategies focusing on health-related initiatives and take the lead in implementing these strategies, especially those aligned with the local government’s authority and legislative capacity.
4. Policy formulation: Formulates and executes policies, plans, programmes, and projects that promote community health. They advise relevant authorities on health-related concerns, contributing to the overall well-being of the local population.
5. Regulatory enforcement: Enforces all laws, ordinances and regulations pertaining to public health and recommends necessary ordinances through the local health board, ensuring the preservation of public health. Additionally, they have the authority to recommend the prosecution of any violations of sanitary laws and regulations.
6. Sanitary inspection: Oversees the sanitary inspection of establishments like food-selling businesses and accommodations, ensuring adherence to sanitation standards.
7. Health promotion: Conducts health information campaigns, disseminating vital health knowledge, and providing health intelligence services.
8. Partner coordination: Coordinates with government agencies and non-governmental organisations involved in health services delivery and promotion to enhance the impact of health initiatives and contribute to a more comprehensive approach to public health.
9. Service delivery and disaster response: Provides health services to local communities regularly and supports service delivery during disasters and emergencies.

## The role of local health officers in advancing integration

In response to these challenges and issues of fragmentation, the Philippine Universal Health Care Act was passed into law, reaffirming the country’s long-standing commitment to providing access to high-quality health services and enhancing the quantity, efficiency and quality of health facilities and workers to ensure effective service delivery. The law primarily focuses on two key reform areas: (1) consolidating various local health systems at the provincial level and highly urbanised cities to minimise fragmentation in health service delivery and (2) establishing healthcare provider networks to provide population-based and individual-based services in a coordinated way. The former seeks to reintegrate the country’s highly devolved health governance system to make it more resilient, sustainable and responsive to the needs of the population, while the latter aims to integrate primary, secondary and tertiary care provision through a seamless navigation and coordination system. Both of these reforms are set to happen at the local levels, clearly highlighting the role of local health officers in advancing these objectives (table 1).

**Table 1:** Roles and activities of local health officers in the context of UHC reforms in the Philippines

Key reforms in the UHC	Functions implicated for local health officers	Domain	Illustrative activities
Systems-level integration: Reorganising the local health system into city-wide and province-wide health systems	Governance and leadership	Public health	Engaging in local health board meetings and supporting the development of policies, programmes and resource allocation Forge partnerships with development organisations, NGOs, community-based groups and other partners to leverage resources and technical expertise
	Planning and monitoring	Public health	Develop and implement evidence-based plans in collaboration with stakeholders Monitoring of health programmes and making recommendations for improvement
Service-level integration: Strengthening population-based and individual-based service provision through the healthcare provider network	Health service delivery	Primary care	Conducting regular community health assessments to identify healthcare needs and priority interventions Implementing promotive and preventive programmes and providing essential services including maternal and child health services, family planning and common illnesses Implementing quality improvement initiatives (ie, adherence to clinical guidelines and protocols Supporting accreditation of health facilities
	Disease surveillance	Public health and Primary care	Establish effective disease surveillance system to monitor and report notifiable diseases and public health emergencies Conducting outbreak investigations, contact tracing and implementing infection prevention and control measures
	Advocacy and community empowerment	Public health and Primary care	Organising health campaigns and community outreach programmes on preventive measures and healthy lifestyles Conducting community meetings/dialogues to ensure community perspectives are considered in healthcare initiatives

NGO, non-government organisation; UHC, universal health coverage.

During the 71st national convention of the Association of Municipal Health Officers of the Philippines held in June 2023, the conference’s theme of ‘Redirecting public health system towards UHC’ echoes the call to initiate a transformative shift in the way the country’s health system is structured, organised and operated. The meeting was participated by 620 MHOs, making it one of the largest single gatherings of local health officers nationwide. This redirection is perceived by the local health officers to involve a series of fundamental changes and reforms aligned with the country’s UHC agenda that prioritises the delivery of comprehensive and integrated services so that individuals and communities can access essential health services without facing financial hardship.

## Codesigning the integrated approach to healthcare delivery

**Figure 1:**
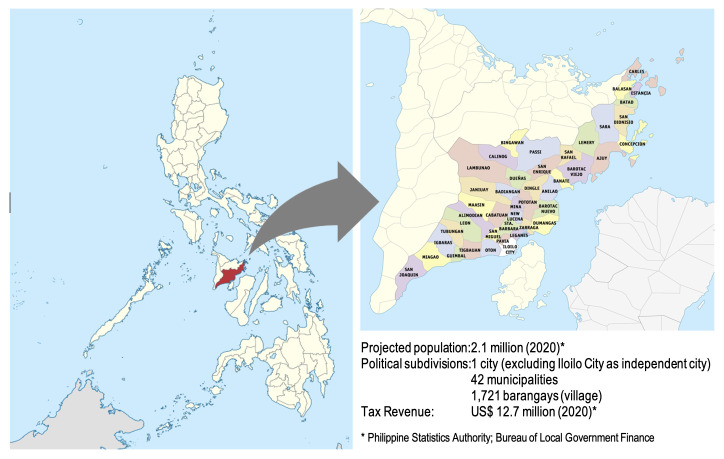
Map of Iloilo province showing geographical and economic profile. Source: OpenStreetMap (https://www.openstreetmap.org/).

Given the prevailing policy context, the Province of Iloilo, with technical support from USAID’s ReachHealth Project (a 6-year health initiative implemented by RTI International aiming to strengthen and improve access to vital health services for Filipino families) has conceptualised an integrated strategy to providing COVID-19, family planning and primary care services. Situated in the southeastern part of the Visayas Islands (one of the main island groups in the central region of the country) (figure 1), the province ranks 14th among 81 provinces in terms of population density and ranks as the 7th wealthiest province in 2022. It was also designated as a UHC integration site (UHC-IS), serving as a demonstration area for modelling the various local health systems reforms outlined in the Universal Health Care law. The UHC-IS approach offers a synergistic method for policy planning and implementation to extract policy-relevant insights.^20^ This approach offers a chance to test out an integrated service delivery mechanism in an area already configured for intervention modelling.

**Figure 2:**
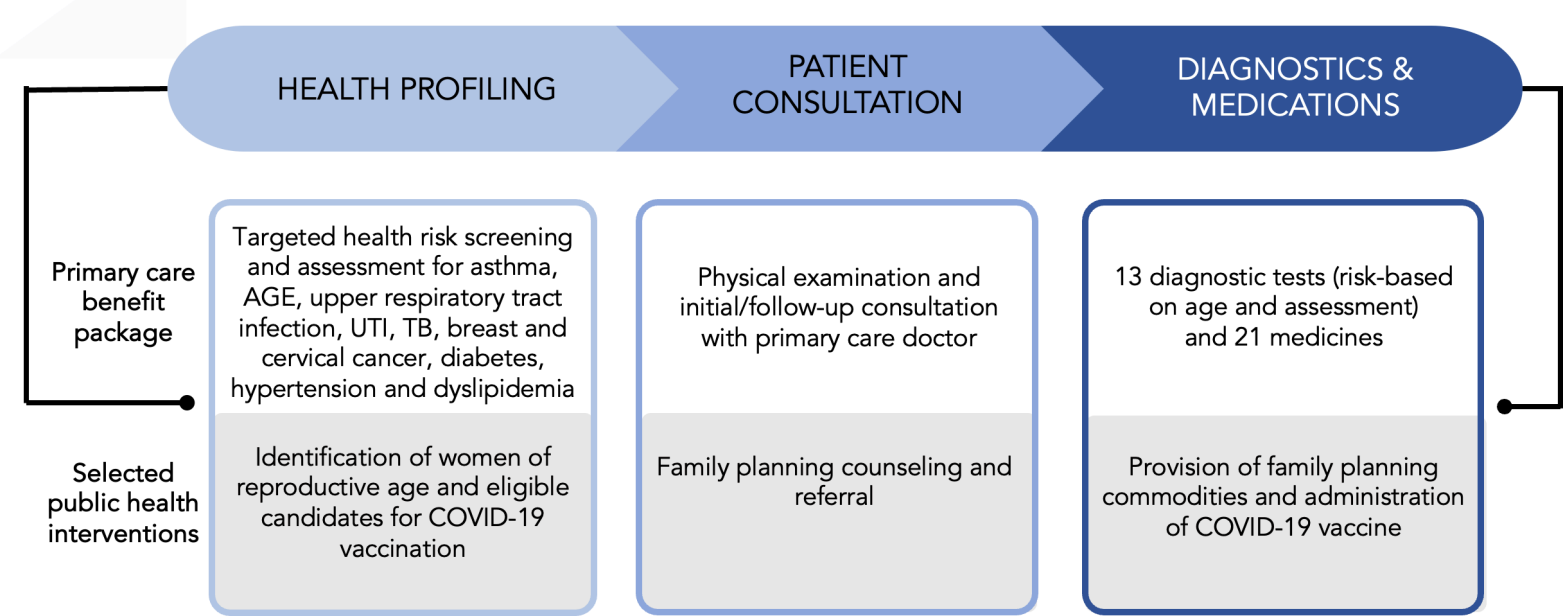
Integrated public health and primary care model in Iloilo province, Philippines. AGE, acute gastroenteritis; TB, tuberculosis; UTI, urinary tract infection.

The initial step taken by the research team was soliciting the interest of local health officers from the 43 component LGUs of the province to codesign and implement an integrated model of health service delivery. Within this framework, our model identified a strategic entry point for incorporating priority public health interventions into the existing primary care benefit structure. The primary care package is disease-agnostic and consists of health profiling, patient consultations, diagnostic tests and medication provisions for selected diseases such as diabetes, hypertension and dyslipidaemia (figure 2). In this collaboration, family planning and COVID-19 vaccination emerged as the priority public health programmes to be integrated. It was established that these programmes held significant importance for the Iloilo province, especially in addressing the need for family planning, which stands at 2.22% among the 522 000 estimated women of reproductive age, closely mirroring the national average of 3.28% in 2021.^21^ Additionally, the pre-intervention COVID-19 vaccination rate in the province, particularly for individuals aged 60 and above, stood at 75.3%, falling short of the national target of 90%.^15^ Acknowledging the critical nature of these public health challenges, the focus was to integrate these interventions into the public health insurance-funded primary care benefit to optimise the efficiency of delivering both vaccination and family planning services and a streamlined approach to healthcare provision.

We capitalised on the established roles of local health officers in both public health and primary care domains to secure their buy-in and ownership of the intervention design. The engagement process generated widespread interest among local health officers fostering strong support for the integration efforts. The participating LGUs were engaged throughout the pilot implementation ensuring that progress was tracked and development of adaptive solutions to challenges was contextually suitable and locally driven. Frequent feedback sessions and evaluations during the mid-implementation and post-implementation phases were carried out to pinpoint factors that supported or hindered the implementation. These efforts yielded valuable observations that were used to enhance the approach, fine-tune the integration design, and propose strategies for scale-up in other sites. However, fully leveraging this opportunity requires further validation and might currently raise uncertainties about its sustainability, particularly in other decentralised contexts and settings that are undergoing epidemiological shifts.

During the 6-month implementation study, the active support of local health officers has been instrumental in executing this integrated approach. Their participation spans from the initial design and planning stages to overseeing medical consultants, engaging in reflective practices and providing valuable input for necessary adjustments. The involvement of local health officers has been a key factor in the successful implementation of the pilot. In the next section, we elaborate on the details of their participation across various capacities, emphasising the indispensable contributions they provide throughout the process.

### Governance and leadership

Local health officers took a hands-on approach to the design, implementation and resource mobilisation of the intervention. They actively participated in the development and fine-tuning of the model and took the lead in its execution by leading the process with their health facility personnel. They also initiated conversations with their local chief executives to advocate for the allocation of supplementary resources including the hiring of data encoders, transport and internet connectivity which were essential for ensuring a seamless implementation of the project.

### Planning and monitoring

Engaging the local health officers in the planning process ensures that the model is tailored to the specific needs and dynamics of each locality. They assumed the responsibility of supervising the medical consultants who were brought in for assistance, performing monitoring of activities and progress, maintaining alignment with the objectives, and verifying that tasks were being carried out according to plan. The diligent review of weekly reports submitted by the consultants allowed for timely feedback and adjustments as needed. Additionally, local health officers actively participated in ‘pause and reflect’ activities, requiring them, to step back from the implementation process at intervals to assess progress and outcomes. During these sessions, they shared insights, discussed challenges and provided valuable suggestions for refining the model’s implementation. This reflective approach promoted a continuous cycle of improvement.

### Health service delivery

Local health officers played a crucial role in expediting the accreditation or reaccreditation application of their health facilities for the primary care package with the public health insurance. They supervised and coordinated patient registration efforts encompassing the registration of their constituent population for primary care, and performing vaccination and family planning services.

### Disease surveillance

Disease outbreak management and surveillance are important in safeguarding the health and well-being of the community. While these were not essentially part of the intervention, local health officers took charge of identifying eligible candidates for COVID-19 vaccination and facilitated the actual delivery of the services during the health profiling activities.

### Advocacy and community empowerment

During the process of registration and health profiling, whether carried out at health facilities or through village visits, the local health officers engaged village leaders and community health workers in conducting health education and promotion activities to raise awareness about COVID-19 vaccination, family planning and other preventive health measures. Advocacy efforts focused on educating community members about the benefits and safety of these services, emphasising the importance of taking proactive steps for maintaining healthy behaviours such as getting regular health check-ups and seeking healthcare when needed.

## Patient and public engagement

The codesign process outlined above represents a form of patient and public engagement that seeks to actively involve the public in all phases of research—from planning and design to implementation and dissemination of findings. In this context, the ‘public’ specifically refers to the local health officers of the 43 LGUs and other stakeholders who were acknowledged for their capacity to provide valuable insights, perspectives and priorities. These contributions were instrumental in enhancing the quality and relevance of the research and were solicited and incorporated at every stage of the project’s implementation.

## Lessons learnt

With the progress made towards UHC and the lessons gathered from the COVID-19 experience, governments often talk of adopting a more integrated approach to healthcare. Regrettably, local health officers, despite playing a crucial role, face a lack of essential platforms and resources to fulfil their vital role in promoting health equity and supporting the reforms. It is fostering social participation and systemic coordination among various stakeholders that ensures policies are responsive to the diverse needs of the population. This not only establishes a foundation of trust in policies but also enhances the transparency and accountability of health systems.^22^ Through this implementation research and our interactions with local health officers, we have identified the following lessons that aim not only to navigate potential pitfalls but also serve to guide the formation of strong partnerships that fully use the unique strengths of this indispensable stakeholder.

### Enhancing capacity and skills to implement integration

One of the risks in enhancing integration is placing an additional burden on already limited resources including human, financial and other assets. To tackle this challenge, it is imperative to align the structure and staffing requirements of local health systems in a manner that actively supports the implementation of integrated practices.^23 24^ This might require reassessing the allocation of health personnel to achieve a balanced distribution of workloads, adjusting scopes of practice and job descriptions using established methodologies like WHO’s workload indicators of staffing needs,^25^ and optimising various processes to achieve cost and financing efficiencies. It is also important to emphasise the need to strengthen the skills of local health officers to effectively carry out their responsibilities within an integrated healthcare setup. Providing capacity-building support for current officers while preparing future practitioners for this role holds great importance in sustaining the reform.^26 27^

### Aligning incentives and allocating resources

Consider realigning financial incentives to attract and retain physicians within the public healthcare system particularly at primary care.^28^ Addressing perverse financing that creates disparity in the way primary care and specialists are paid in many health systems is an important step.^29^ By creating a more equitable and attractive financial environment for local health officers, it can foster a stronger emphasis on preventive measures and timely interventions. Such shifts can potentially reduce the burden of expensive and complex treatments that stem from health issues being addressed belatedly or inadequately managed. Providing appropriate salary structure and competitive remuneration, as well as standardising job positions, fosters a more attractive and equitable environment that encourages skilled healthcare practitioners to gravitate towards public primary care and remain committed to its objectives emerges as pivotal strategies.

### Building trust and fostering collaboration

Partnerships with clear articulation of roles and functions are key for national health agencies (ie, ministry of health and public health insurance) to engender the trust of local policymakers and health officers to work towards integration.^30^ Setting shared objectives and creating open and transparent channels for communicating collective learning and addressing challenges collaboratively can promote understanding, trust and respect. This may involve the orchestration of local intersectoral bodies, bringing together diverse stakeholders to the task. Collaborative frameworks such as the Context and Capabilities for Integrating Care^31^ provide a useful means to further define the capacity needs of local actors and enhance the cooperative processes. Consequently, there arises the need to bolster governance mechanisms for accountability, performance management and cooperation among all involved partners. This is crucial in maintaining transparency and driving forward successful integrated healthcare initiatives.

### Establishing IT-enabled monitoring and evaluation system

Regularly assessing outcomes helps identify areas for enhancement thereby enabling adjustments in interventions in both structure and processes of collaboration. The use of information technology is crucial to support local health officers in the monitoring and evaluation of integration efforts.^32^ Primarily, it is necessary to appraise community health needs through data aggregation and analysis to identify health trends, potential outbreaks and health-compromising exposures at the community level.^29^ This will empower local health systems to proactively address disease prevention and early intervention needs. Technology can also play a vital role in assessing the responsiveness of interventions at the primary care level, enhancing care coordination between primary care physicians and specialists, minimising redundant and conflicting services, and ensuring that patients are receiving the right care at the right time from the most appropriate provider.

In the Philippines, as in other decentralised settings, pursuing the integration of health services emphasises the significance of the human aspect alongside the necessary policy and structural changes. While the PHC framework advocates for integration-focused policies, it also underscores the importance of leveraging existing organisational structures and respecting governance arrangements within a multisectoral set-up as necessary implements for facilitating integration.^33^ Our findings align with recent studies highlighting the crucial role of supportive structures, policy coherence and effective managerial-health profession relations.^34 35^ Through our interactions with the local health officers, a collaborative approach emerges, fostering coproduction, shared responsibility and accountability—values that underpin successful integration.^36^ This underscores the need to not only implement policy changes but also to actively engage with the individuals and organisations involved, recognising the intricate interplay between human dynamics and structural adjustments in achieving integrated health services.

## Conclusion

The integration of primary care and public health domains is undeniably shaped by policy initiatives at the national level and corresponding responses from local governance, resulting in pragmatic care designs that reflect contextual factors and local competence. In this intricate interplay, local health officers emerge as central figures, playing a pivotal role in enabling this interaction and bridging the gap between promotive, preventive and curative aspects of healthcare. Perhaps one of the transformative roles they play is in fostering effective collaboration across various stakeholders through their understanding of both healthcare and community dynamics. Therefore, an integrated approach that harnesses the expertise of local health officers to facilitate improved health education, preventive measures, targeted interventions and effective intersectoral collaboration will ultimately empower the delivery of comprehensive and holistic healthcare services to communities and foster better health outcomes for all.

## Data Availability

No data are available.
